# Comment on: Portal vein embolization *versus* dual vein embolization for management of the future liver remnant in patients undergoing major hepatectomy: meta-analysis

**DOI:** 10.1093/bjsopen/zrae057

**Published:** 2024-07-04

**Authors:** Hani Oweira, Bassem Krimi, Amine Gouader, Ian Seiller, Mohamed Ali Chaouch

**Affiliations:** Department of Surgery, Universitätsmedizin Mannheim, Heidelberg University, Mannheim, Germany; Department of General Surgery, Perpignan Hospital, Perpignan, France; Department of General Surgery, Perpignan Hospital, Perpignan, France; Department of Radiology, Perpignan Hospital, Perpignan, France; Department of Visceral and Digestive Surgery, Fattouma Bourguiba Hospital, University of Monastir, Monastir, Tunisia

The systematic review and meta-analysis by Bell *et al*.^1^ is of great interest and they are congratulated for their valuable study. However, the following comments on the results of the study are offered.

**Fig. 1:**
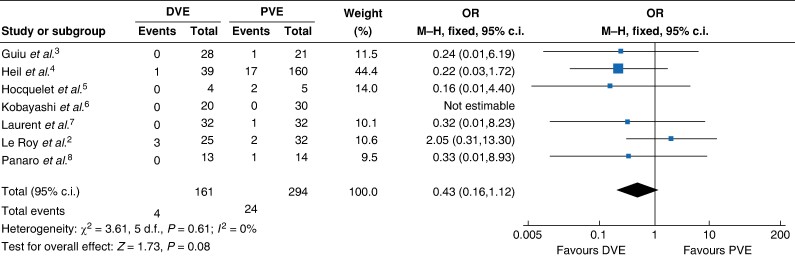
Corrected forest plot of mortality rate

DVE, dual vein embolization; PVE, portal vein embolization.

Bell *et al*.^1^ concluded that dual vein embolization was associated with a lower mortality rate compared with portal vein embolization, as depicted in the forest plot in Figure 4 of their article. However, a significant error in this forest plot erroneously altered the direction of the pooled effect size, creating a false impression of a beneficial effect on mortality rates. Specifically, the reported number of events in the two groups from the study by Le Roy *et al*.^2^ was inaccurate. The number of mortalities in the dual vein embolization group should be three, not one. Upon re-analysing the data using the fixed-effect model, there is no significant difference between the two groups (OR 0.43, 95% c.i. 0.16 to 1.12; *P* = 0.080) (*Fig. 1*), with no heterogeneity among the studies.

In conclusion, contrary to the findings of Bell *et al*.^1^, the corrected analysis of the available evidence does not support the superiority of dual vein embolization over portal vein embolization in reducing mortality in major hepatectomy. It is crucial to present this corrected finding to the readers of *BJS Open* to avoid misinterpretation of these results.
